# The multifaceted role of SQSTM1/p62 in disc degeneration: A master regulator of cellular stress responses

**DOI:** 10.1016/j.bbrep.2025.102222

**Published:** 2025-08-29

**Authors:** Yang Hou, Lei Liu, Tianyi Zhao, Yongfei Guo, Jiangang Shi

**Affiliations:** Department of Orthopaedic Surgery, Changzheng Hospital, Second Military Medical University, No. 415 Fengyang Rd, Shanghai, 200003, China

**Keywords:** Sequestosome 1 (SQSTM1/p62), Intervertebral disc degeneration (IDD), Autophagy, Oxidative stress, Inflammation

## Abstract

Intervertebral disc degeneration (IDD) is a key contributor to lumbar degenerative diseases and chronic low back pain. Accumulating evidence indicates that Sequestosome 1 (SQSTM1/p62), a multifunctional adaptor protein, plays a pivotal role in IDD pathogenesis through its regulation of autophagy, oxidative stress, inflammation, and programmed cell death. This review summarizes the multifaceted functions of SQSTM1 in the context of IDD, including its involvement in the autophagy-lysosome pathway, antioxidant defense via the Keap1-Nrf2 axis, activation of the NF-κB signaling and NLRP3 inflammasome, and modulation of apoptosis, pyroptosis, and ferroptosis. Moreover, SQSTM1 contributes to extracellular matrix degradation by upregulating matrix metalloproteinases and downregulating their inhibitors. Given its dynamic expression during disc degeneration, SQSTM1 holds promise as both a biomarker for IDD progression and a therapeutic target. Potential strategies targeting SQSTM1 include the use of autophagy inducers, inflammatory pathway inhibitors, and ferroptosis/pyroptosis modulators. However, challenges remain in precisely modulating SQSTM1 activity and translating findings into clinical therapies. Future research leveraging advanced technologies such as single-cell RNA sequencing, proteomics, and organoid models is essential to unravel the complex, stage- and cell-specific roles of SQSTM1 in IDD. Understanding these mechanisms may open new avenues for effective treatment and improved patient outcomes in degenerative spinal disorders.

## Highlights

• SQSTM1/p62 links autophagy, oxidative stress, and inflammation in disc degeneration.
• Abnormal SQSTM1 drives apoptosis, pyroptosis, and ferroptosis in NP and AF cells.
• SQSTM1 promotes ECM degradation by modulating MMPs and TIMPs expression.
• Targeting SQSTM1 may alleviate IDD via autophagy activation or inflammation inhibition.
• SQSTM1 is a potential biomarker and therapeutic target for intervertebral disc disorders.

## Graphical abstract

**Figure:**
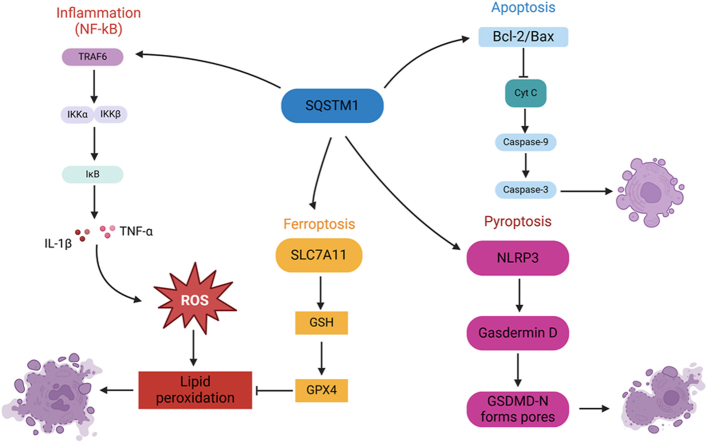

## 1 Introduction

Intervertebral disc degeneration (IDD) is a major pathological process underlying lumbar degenerative diseases (LDDs) and chronic low back pain. Epidemiological studies indicate that the incidence of IDD increases significantly with age and is closely associated with genetic predisposition, mechanical stress, inflammation, and oxidative stress [[1], [2], [3]]. The pathological features of IDD primarily include cellular apoptosis, autophagy imbalance, inflammatory responses, pyroptosis, ferroptosis, and extracellular matrix (ECM) degradation [4,5].

During IDD progression, the dysfunction of nucleus pulposus (NP) cells, annulus fibrosus (AF) cells, and cartilaginous endplate (CEP) cells plays a crucial role. In the early stages, IDD is characterized by metabolic imbalances in NP cells, leading to decreased ECM synthesis and increased inflammatory cytokine release [6,7]. As the degeneration advances, excessive activation of apoptosis and ferroptosis further exacerbates ECM degradation, ultimately resulting in structural and functional failure of the intervertebral disc [8,9]. Moreover, alterations in the disc microenvironment, such as increased oxidative stress and nutrient deficiency, contribute to disease progression [10]. While current treatment strategies for IDD mainly involve conservative management and surgical intervention, reversing the degeneration remains a significant challenge. Therefore, elucidating the molecular mechanisms of IDD and identifying potential therapeutic targets are of paramount importance.

**Fig. 1:**
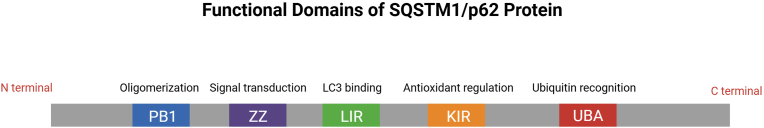
Functional domains of the SQSTM1/p62 protein and their associated cellular roles.

Sequestosome 1 (SQSTM1), also known as p62, is a multifunctional adaptor protein involved in autophagy, protein degradation, and signal transduction [11,12]. Structurally, SQSTM1 contains several functional domains, including the PB1 domain, which mediates protein oligomerization; the UBA domain, which binds ubiquitinated proteins [12,13]; the LIR motif, which interacts with LC3 to facilitate autophagic degradation [14]; and the KIR domain, which regulates the Keap1-Nrf2 antioxidant signaling pathway [15], (Fig. 1).

**Fig. 2:**
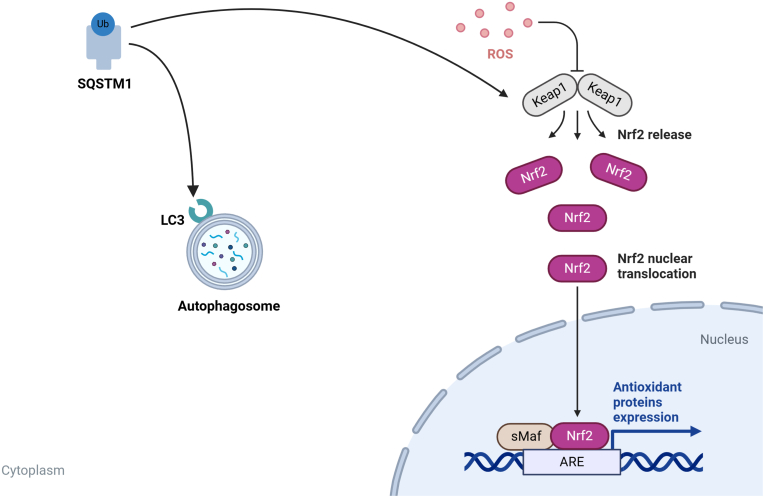
SQSTM1-mediated regulation of autophagy and antioxidant response via the Keap1-Nrf2 pathway.

SQSTM1 plays a critical role in maintaining cellular homeostasis through multiple mechanisms. It functions as a selective autophagy receptor, targeting ubiquitinated proteins for degradation via autophagosome-lysosome pathways [16]. Additionally, it regulates the NF-κB signaling cascade by interacting with TRAF6, thereby promoting inflammatory responses [17]. Moreover, SQSTM1 modulates oxidative stress through the Keap1-Nrf2 axis, where it competes with Keap1 to release Nrf2, leading to the transcriptional activation of antioxidant genes [18,19], (Fig. 2). Furthermore, SQSTM1 is involved in the ubiquitin-proteasome system, contributing to protein turnover and degradation [20,21].

Recent studies have highlighted the involvement of SQSTM1 in various diseases, including neurodegenerative disorders, cancer, and musculoskeletal diseases [12,22]. However, its precise role in IDD remains incompletely understood. Emerging evidence suggests that SQSTM1 influences IDD progression through mechanisms related to autophagy dysregulation, inflammation, oxidative stress, and cell death. Therefore, investigating the role of SQSTM1 in IDD could provide novel insights into its molecular pathology and pave the way for targeted therapeutic strategies.

Compared to classical autophagy-related proteins such as Beclin1, LC3, and ATG family members, which mainly function within the autophagosome formation machinery, SQSTM1 serves a broader integrative role across multiple cellular stress response pathways [16,23]. Specifically, SQSTM1 not only mediates selective autophagy but also directly interfaces with oxidative stress via the Keap1-Nrf2 axis and modulates inflammation and cell death through interactions with TRAF6 and caspase-related signaling [16]. This unique cross-functional involvement positions SQSTM1 as a master regulator or “central hub” that coordinates cellular fate decisions under degenerative stress. Emphasizing its versatility may provide new insights into the pathophysiology of IDD.

## 2 The role and mechanism of SQSTM1 in intervertebral disc degeneration (IDD)

Recent studies suggest that abnormal SQSTM1 expression may accelerate IDD progression. The following sections discuss the mechanisms by which SQSTM1 influences different pathological aspects of IDD.

### 2.1 SQSTM1 and autophagy-lysosome pathway regulation

Autophagy is essential for maintaining cellular homeostasis and survival. In IDD, autophagy levels in nucleus pulposus (NP) and annulus fibrosus (AF) cells progressively decline, leading to the accumulation of damaged proteins and organelles, thereby accelerating cellular dysfunction and degeneration.

SQSTM1 is a key regulator of autophagy and affects IDD progression through multiple mechanisms. As a selective autophagy receptor, SQSTM1 binds to ubiquitinated proteins and interacts with LC3 via its LC3-interacting region (LIR), directing damaged proteins toward autophagic degradation [24]. However, abnormal SQSTM1 accumulation may result in autophagic flux blockade, preventing the effective clearance of toxic cellular components [25]. Additionally, SQSTM1 interacts with the mTORC1 complex, influencing mTOR activation and autophagy initiation. Dysregulated SQSTM1 expression may lead to persistent mTORC1 activation, thereby inhibiting autophagy and exacerbating IDD [26,27]. Moreover, SQSTM1 plays a role in mitophagy by interacting with Parkin to facilitate the clearance of damaged mitochondria [28]. In IDD, dysregulation of SQSTM1 leads to impaired mitophagy, which not only exacerbates oxidative stress but also induces secondary activation of the NLRP3 inflammasome through ROS accumulation, thereby triggering chronic inflammatory responses and cell death [29,30], highlighting its pivotal role as a hub connecting autophagy, oxidative stress, and inflammation.

### 2.2 SQSTM1 and oxidative stress

Oxidative stress plays a critical role in IDD progression, primarily caused by excessive accumulation of reactive oxygen species (ROS) [2,31]. SQSTM1 regulates antioxidant defense through the Keap1-Nrf2 signaling axis. By binding to Keap1 via its Keap1-interacting region (KIR), SQSTM1 promotes Keap1 degradation, leading to Nrf2 activation. Activated Nrf2 translocates into the nucleus, inducing the transcription of antioxidant genes such as HO-1, NQO1, and SOD, thereby enhancing cellular defense against oxidative damage [18,32]. However, in IDD, aberrant expression of SQSTM1 may impair Nrf2 activity, reduce antioxidant capacity, and exacerbate ROS accumulation. As a danger signal, ROS can further activate the NLRP3 inflammasome, promoting chronic inflammation and programmed cell death, thereby forming a vicious cycle [33]. Additionally, dysfunctional mitophagy due to SQSTM1 dysregulation may lead to mitochondrial dysfunction and excessive ROS production, further accelerating IDD progression through oxidative damage and inflammatory responses [34].

### 2.3 SQSTM1 and inflammatory responses

Chronic inflammation is a hallmark of IDD. SQSTM1 plays a crucial role in regulating inflammation via the NF-κB signaling pathway and the NLRP3 inflammasome [35]. SQSTM1 interacts with TRAF6 to enhance IKK complex activation, leading to NF-κB nuclear translocation and upregulation of pro-inflammatory cytokines such as IL-1β, TNF-α, and IL-6. Persistent NF-κB activation driven by abnormal SQSTM1 accumulation can sustain inflammation, contributing to disc degeneration [36,37].

In addition, SQSTM1 regulates the activation of the NLRP3 inflammasome, which is a critical step for the maturation and release of IL-1β and IL-18 [38]. This process is closely associated with SQSTM1-mediated autophagy imbalance: autophagy impairment-induced SQSTM1 aggregation and mitochondrial damage are key triggers for inflammasome activation, which in turn further amplify pyroptosis and other forms of inflammatory cell death. Dysregulated SQSTM1 expression may enhance NLRP3 activation, thereby amplifying inflammatory responses in IDD. In the inflammatory microenvironment of IDD, SQSTM1 promotes M1 macrophage polarization, increasing the secretion of pro-inflammatory mediators and exacerbating tissue damage [39].

### 2.4 SQSTM1 and cell death (apoptosis, pyroptosis, ferroptosis)

Cell death is a critical process in IDD, and SQSTM1 is involved in multiple cell death pathways, including apoptosis, pyroptosis, and ferroptosis. SQSTM1 influences apoptosis by regulating the Bcl-2 family proteins (Bax/Bcl-2) and activating the JNK signaling pathway [[40], [41], [42]]. Overexpression of SQSTM1 may enhance JNK activity, promoting apoptosis and accelerating IDD progression. Pyroptosis, a form of inflammatory cell death, is also linked to SQSTM1, as it facilitates Gasdermin D (GSDMD) cleavage and activation, leading to the release of pro-inflammatory cytokines and further tissue damage [37,43].

Ferroptosis, an iron-dependent form of cell death, has recently been implicated in IDD. SQSTM1 regulates ferroptosis by modulating GPX4 and SLC7A11 expression. Abnormal SQSTM1 accumulation may suppress GPX4 activity, leading to lipid peroxidation and ferroptosis, thereby promoting disc degeneration [[44], [45], [46]].

Beyond its well-characterized roles in apoptosis, pyroptosis, and ferroptosis, recent studies have revealed that SQSTM1 also modulates alternative cell death pathways such as necroptosis and PANoptosis. SQSTM1 can regulate caspase-8 activity, a key mediator that integrates apoptotic and necroptotic signaling. Under certain stress conditions, p62/SQSTM1 recruits caspase-8 into protein aggregates or autophagosomes, modulating its activation status and thereby influencing downstream cell fate decisions [47,48]. Moreover, SQSTM1 has been implicated in PANoptosis, a form of inflammatory cell death that integrates pyroptosis, apoptosis, and necroptosis, through its interaction with key regulators such as RIPK1, RIPK3, and CASP8. These findings suggest that SQSTM1 serves not only as an autophagy adaptor but also as a central scaffold protein that orchestrates multi-modal programmed cell death (PCD) pathways in response to cellular stress [49]. Its context-dependent role in regulating these complex death mechanisms may be particularly relevant in the inflammatory microenvironment of IDD, where overlapping forms of cell death contribute to disc degeneration.

### 2.5 SQSTM1 and extracellular matrix metabolism

ECM degradation is a key feature of IDD, and SQSTM1 is involved in regulating ECM turnover. SQSTM1 modulates the balance between matrix metalloproteinases (MMPs) and their inhibitors (TIMPs). Upregulation of SQSTM1 enhances NF-κB signaling, leading to increased MMP expression and subsequent ECM degradation. Simultaneously, SQSTM1 downregulates TIMP-1/2 expression, further disrupting ECM homeostasis [50,51].

Moreover, SQSTM1 interacts with various signaling pathways, including Wnt/β-catenin and MAPK, which are involved in ECM remodeling [52,53]. Its dysregulation may contribute to excessive ECM breakdown by activating catabolic enzymes while suppressing anabolic factors. The loss of ECM integrity weakens the biomechanical properties of the intervertebral disc, making it more susceptible to degeneration and structural collapse. As a result, SQSTM1 plays a pivotal role in maintaining ECM homeostasis, and its abnormal expression may accelerate IDD progression by tipping the balance toward matrix degradation.

## 3 The potential clinical applications of SQSTM1 in IDD

Recent studies have highlighted the significant role of SQSTM1 in autophagy, inflammation, and cell death, making it a promising candidate for clinical applications in intervertebral disc degeneration (IDD). SQSTM1 may serve as both a biomarker for disease progression and a potential therapeutic target for IDD intervention.

### 3.1 SQSTM1 as a potential biomarker

SQSTM1 expression is dynamically altered in degenerative intervertebral disc tissues, with evidence suggesting its upregulation in later stages of IDD [54]. This expression pattern may correlate with disease severity and progression, particularly in relation to autophagy dysfunction. Additionally, studies indicate that SQSTM1 levels could be associated with Modic changes and inflammation in IDD [55,56]. Evaluating SQSTM1 expression in intervertebral disc tissue might provide insights into disease progression.

Moreover, SQSTM1 may serve as a circulating biomarker for IDD. Soluble SQSTM1 in serum, cerebrospinal fluid, or extracellular vesicles could be detected using enzyme-linked immunosorbent assays (ELISA) or mass spectrometry, offering a non-invasive diagnostic tool. Further studies are required to determine the specificity and sensitivity of SQSTM1 as a biomarker for IDD.

### 3.2 Therapeutic strategies targeting SQSTM1

Since SQSTM1 plays a central role in autophagy regulation, inflammation, and programmed cell death, modulating its activity presents a potential therapeutic avenue for IDD. Recent studies have demonstrated that autophagy protects against LPS-induced nucleus pulposus (NP) cell pyroptosis via a P62/SQSTM1-mediated degradation mechanism, and inhibiting pyroptosis can slow IDD progression in vivo [57]. This highlights the potential of targeting SQSTM1 in IDD treatment. Additionally, research has shown that autophagy is negatively regulated by the PI3K/Akt/mTOR signaling pathway, which plays a crucial role in maintaining disc cell function. Rapamycin, an mTORC1 inhibitor, has been found to induce autophagy in annulus fibrosus (AF) cells, as evidenced by increased LC3-II expression and reduced SQSTM1 levels, thereby alleviating IL-1β-induced inflammation, apoptosis, and extracellular matrix degradation. However, the protective effects of rapamycin are dependent on Akt and mTORC2 activity, further underscoring the complex regulation of autophagy in IDD [50].

Targeting SQSTM1-mediated inflammation may also offer therapeutic benefits. As SQSTM1 is a key regulator of NF-κB signaling, NF-κB inhibitors such as BAY 11–7082 may reduce SQSTM1-induced inflammatory cytokine release and chronic inflammation in IDD. Furthermore, modulating macrophage polarization by targeting SQSTM1 may influence the inflammatory microenvironment and mitigate IDD progression.

SQSTM1 is also implicated in ferroptosis and pyroptosis, both of which contribute to IDD pathogenesis. Ferroptosis inhibitors such as ferrostatin-1 could help protect disc cells from SQSTM1-mediated oxidative damage, while pyroptosis inhibitors such as MCC950 could regulate the SQSTM1/NLRP3 axis to suppress sterile inflammation in IDD.

Despite the therapeutic potential of targeting SQSTM1 in IDD, several critical challenges remain. First, achieving target specificity is a major hurdle. Given the ubiquitous expression and pleiotropic functions of SQSTM1 in various tissues, systemic administration of SQSTM1-targeted agents may result in off-target effects, including unintended modulation of immune responses, metabolic pathways, or autophagy in non-disc tissues. This underscores the need for precision delivery strategies that selectively target intervertebral disc cells while minimizing systemic exposure. Recent advances in nanoparticle-based delivery systems offer promising solutions for disc-specific targeting. For instance, injectable nanoparticles functionalized with ECM-binding peptides, hyaluronic acid coatings, or cell-penetrating ligands have been developed to deliver siRNA, CRISPR components, or small molecules specifically to nucleus pulposus or annulus fibrosus cells. These platforms can enhance therapeutic payload accumulation within degenerated discs while reducing systemic toxicity. Moreover, stimuli-responsive nanocarriers, activated by pH, ROS, or enzymatic microenvironments within degenerating discs, may further improve the precision of SQSTM1 modulation. However, these technologies are still in preclinical stages and require rigorous evaluation of their biocompatibility, immunogenicity, and long-term safety. Future studies should therefore prioritize the development and validation of disc-specific delivery systems as a key step toward translating SQSTM1-targeted therapies into clinical practice.

## 4 Future research directions and perspectives

Despite growing evidence supporting the role of SQSTM1 in intervertebral disc degeneration (IDD), several challenges remain in fully elucidating its mechanisms and translating these findings into clinical applications. Current research is largely based on in vitro models and animal studies, which do not fully capture the complexity of human IDD. While in vitro studies help clarify SQSTM1's molecular functions, they lack the physiological context of the intervertebral disc microenvironment. Animal models, though valuable, do not perfectly mimic the progressive and multifactorial nature of human IDD [58,59]. Additionally, the lack of large-scale clinical data limits our understanding of SQSTM1's expression patterns in IDD patients and its potential as a diagnostic or prognostic marker. Another critical gap is the unclear temporal dynamics of SQSTM1 in IDD progression. Whether its effects are primarily protective or pathogenic at different stages remains unresolved, making it essential to define its role in early versus late-stage degeneration.

Notably, SQSTM1 not only participates individually in processes such as autophagy, oxidative stress, inflammation, and cell death, but also orchestrates the cross-regulatory network among them. For example, impaired autophagy leads to ROS accumulation, which subsequently activates inflammasomes and cell death pathways. This multilayered integrative function underscores the pivotal role of SQSTM1 as a central hub in the pathological progression. Beyond its well-established functions in autophagy and oxidative stress, SQSTM1 likely interacts with other key signaling pathways relevant to IDD pathology. The Wnt/β-catenin pathway, known for its role in extracellular matrix homeostasis, may be influenced by SQSTM1 through its interaction with Dishevelled proteins. Similarly, the mTOR pathway, a central regulator of cell metabolism and autophagy, could be modulated by SQSTM1 in ways that impact cell survival and inflammation within degenerating discs. Furthermore, SQSTM1's involvement in NF-κB and MAPK signaling suggests it plays a role in IDD-associated inflammation, potentially amplifying chronic inflammatory responses that contribute to disease progression [60,61]. Understanding these interactions will be crucial for identifying new therapeutic targets and intervention strategies.

Importantly, SQSTM1 exhibits a distinct “double-edged sword” characteristic during the progression of IDD. In the early stages, moderate upregulation of SQSTM1 enhances autophagy and activates the Keap1-Nrf2 antioxidant pathway, offering cytoprotective effects by mitigating oxidative stress and preserving cellular homeostasis [62]. However, in the late stages of IDD, persistent or excessive accumulation of SQSTM1 may lead to autophagic flux impairment, mitochondrial dysfunction, and excessive activation of inflammatory and cell death pathways—such as NF-κB signaling, pyroptosis, and ferroptosis—thereby exacerbating disc degeneration [63,64]. This stage-dependent functional switch highlights the necessity of temporally and spatially controlled SQSTM1 modulation as a therapeutic strategy in IDD management.

Moreover, its function is likely cell type-specific, differing among nucleus pulposus cells, annulus fibrosus cells, and immune cells within the disc. Recent evidence suggests that the functions of SQSTM1 exhibit notable cell type-specific differences within the intervertebral disc. In nucleus pulposus (NP) cells, SQSTM1 predominantly regulates autophagic flux, mitochondrial quality control, and redox homeostasis; its dysregulation often results in impaired mitophagy, ROS accumulation, and cell death [30]. In contrast, in annulus fibrosus (AF) cells, SQSTM1 appears to more strongly modulate inflammation-related pathways, such as NF-κB and MAPK, thereby influencing extracellular matrix turnover and apoptotic responses [65]. In immune cells, particularly macrophages, SQSTM1 regulates polarization toward M1 or M2 phenotypes, shaping the inflammatory milieu through modulation of cytokine secretion and inflammasome activation [66]. These mechanistic differences underscore the need to delineate the spatiotemporal expression and functional roles of SQSTM1 across distinct cell populations, which may guide more precise and cell-targeted therapeutic strategies in IDD. Future studies should investigate these context-dependent effects to clarify whether SQSTM1 should be targeted for inhibition or activation in different disease stages.

While SQSTM1 presents an attractive target for IDD treatment, several challenges must be addressed before clinical translation. Precision modulation of its activity is critical, as both excessive and insufficient expression could have adverse consequences. Small-molecule inhibitors, RNA interference, or CRISPR-based gene editing may provide targeted ways to regulate SQSTM1 function. However, since SQSTM1 is involved in multiple physiological processes beyond IDD, systemic inhibition could lead to unintended side effects such as immune dysregulation or metabolic disturbances. Strategies to enhance tissue specificity, such as nanoparticle-based drug delivery or bioengineered scaffolds, may help mitigate these risks. Furthermore, combining SQSTM1-targeted therapies with existing IDD treatments—such as platelet-rich plasma, stem cell therapy, or biomaterial-based interventions—may enhance regenerative potential and improve therapeutic outcomes.

Advancements in research technologies will be instrumental in deepening our understanding of SQSTM1 in IDD. Single-cell RNA sequencing can provide insights into its expression across different cell populations, while spatial transcriptomics can reveal its distribution within degenerating discs. Proteomics and metabolomics approaches may uncover novel regulatory interactions, and the use of organoid models or 3D bioprinting could offer more physiologically relevant platforms for evaluating potential therapies. As these technologies evolve, they will allow for more precise investigation of SQSTM1's role in IDD and its potential as a therapeutic target.

Overall, SQSTM1 represents a promising but complex player in IDD pathophysiology. While its involvement in autophagy, inflammation, oxidative stress, and cell death is increasingly recognized, many questions remain regarding its precise function across different disease stages and cell types. With continued advancements in molecular biology, bioengineering, and targeted therapies, SQSTM1-based interventions may one day provide new strategies for slowing or even reversing intervertebral disc degeneration, ultimately improving patient outcomes in degenerative spinal disorders.

## CRediT authorship contribution statement

**Yang Hou:** Writing – original draft. **Lei Liu:** Writing – review & editing. **Tianyi Zhao:** Supervision. **Yongfei Guo:** Writing – review & editing. **Jiangang Shi:** Conceptualization.

## Declaration of competing interest

The authors declare no competing interests.

## Data Availability

No data was used for the research described in the article.
